# 1289. The Challenge of Treating Community-Associated Enterobacterales Infections in a Middle-Income Country: Data from SMART 2018-2019

**DOI:** 10.1093/ofid/ofab466.1481

**Published:** 2021-12-04

**Authors:** João Paulo Telles, Lavinia Arend, Larissa Bail, Carmen Ito, Felipe Tuon

**Affiliations:** 1 Pontifical Catholic University - School of Medicine, Curitiba, Parana, Brazil; 2 Universidade Católica do Paraná, São Paulo, Sao Paulo, Brazil

## Abstract

**Background:**

Antimicrobial stewardship programs have been used widely in hospital settings due to the rise of resistant bacteria, antibiotic toxicities, and costs. Nevertheless, few efforts are done to prevent the rising antimicrobial resistance in community settings. The aim of our study was to evaluate the antimicrobial resistance from Enterobacterales community- and hospital-acquired infections in Southern Brazil.

**Methods:**

A total of 272 Enterobacterales isolates (i.e., *Escherichia coli*, *Klebsiella* spp., *Citrobacter* spp., *Enterobacter* spp., *Serratia* spp., *Proteus* spp., and *Providencia*) were collected from 2018 and 2019. Broth microdilution method was used to determine minimum inhibitory concentrations for ceftriaxone, cefepime, levofloxacin, amikacin and ertapenem. Molecular evaluation of beta-lactamases (ESBLs, AmpC, and KPC) was also performed.

**Results:**

Ninety-three, and a hundred and seventy-nine isolates were from community- and hospital-acquired infections, respectively. Similar MIC distribution was found between community and hospital settings (Table 1). Levofloxacin MIC of 8mg/L occurred in 38.7% (n=36) and 30.7% (n=55) of isolates from community- and hospital-acquired infections, respectively (Figure 1). Ceftriaxone MIC of 16mg/L occurred in 39.7%(n=37) and 39.1% (n=70) of isolates from community- and hospital-acquired infections, respectively (Figure 1). At last, cefepime MIC of 32mg/L occurred in 22% (n=21) and 25% (n=46) of isolates from community- and hospital-acquired infections, respectively. The following beta-lactamases were found in isolates from community-acquired group, ACT-MIR, CTX-M, SHV and TEM; while beta-lactamases from the hospital-acquired group were ACT-MIR, CMY II, KPC-2, CTX-M, SHV and TEM.

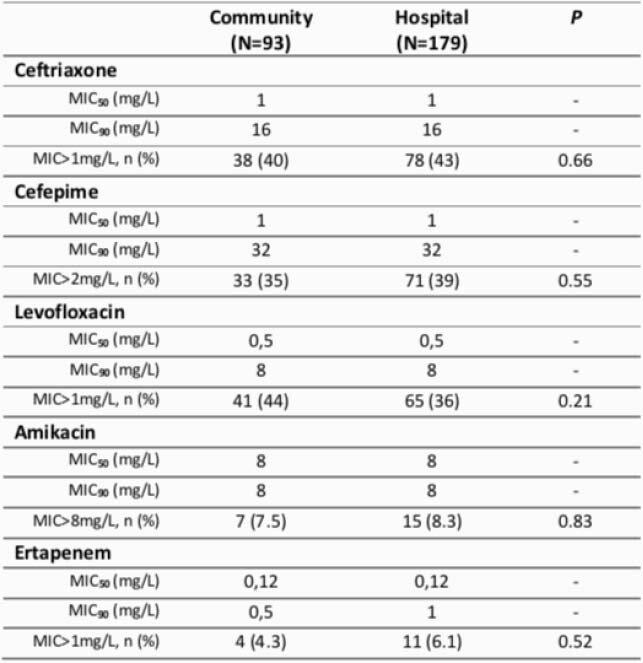

Table 1. Enterobacterales ceftriaxone, cefepime, levofloxacin, amikacin and ertapenem minimum inhibitory concentrations (mg/L) distribution from community- and hospital-settings.

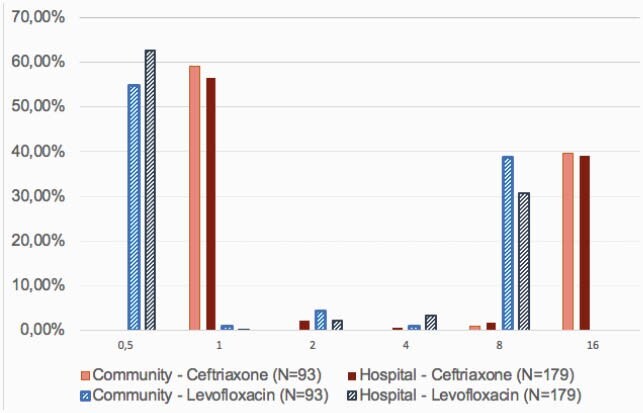

Figure 1. Enterobacterales ceftriaxone and levofloxacin minimum inhibitory concentrations (mg/L) distribution from community- and hospital-settings.

**Conclusion:**

Similar antimicrobials resistances were found in Enterobacterales from community- and hospital-acquired infections. New anti-infective agents are needed urgently to treat pathogens from the community-acquired infections and hospitals that have resistance to the first line regimen. Additionally, community antimicrobial stewardship programs are required.

**Disclosures:**

**All Authors**: No reported disclosures

